# Restriction site-associated DNA sequencing for SNP discovery and high-density genetic map construction in southern catfish (*Silurus meridionalis*)

**DOI:** 10.1098/rsos.172054

**Published:** 2018-05-30

**Authors:** Mimi Xie, Yao Ming, Feng Shao, Jianbo Jian, Yaoguang Zhang, Zuogang Peng

**Affiliations:** 1 Key Laboratory of Freshwater Fish Reproduction and Development (Ministry of Education), Southwest University School of Life Sciences, Chongqing 400715, People's Republic of China; 2 BGI Genomics, BGI-Shenzhen, Shenzhen 518083, People's Republic of China

**Keywords:** catfish, genetic map, single-nucleotide polymorphism, restriction site-associated DNA sequencing

## Abstract

Single-nucleotide polymorphism (SNP) markers and high-density genetic maps are important resources for marker-assisted selection, mapping of quantitative trait loci (QTLs) and genome structure analysis. Although linkage maps in certain catfish species have been obtained, high-density maps remain unavailable in the economically important southern catfish (*Silurus meridionalis*). Recently developed restriction site-associated DNA (RAD) markers have proved to be a promising tool for SNP detection and genetic map construction. The objective of the present study was to construct a high-density linkage map using SNPs generated by next-generation RAD sequencing in *S. meridionalis* for future genetic and genomic studies. An F1 population of 100 individuals was obtained by intraspecific crossing of two wild heterozygous individuals. In total, 77 634 putative high-quality bi-allelic SNPs between the parents were discovered by mapping the parents' paired-end RAD reads onto the reference contigs from both parents, of which 54.7% were transitions and 45.3% were transversions (transition/transversion ratio of 1.2). Finally, 26 714 high-quality RAD markers were grouped into 29 linkage groups by using de novo clustering methods (Stacks). Among these markers, 4514 were linked to the female genetic map, 23 718 to the male map and 6715 effective loci were linked to the integrated map spanning 5918.31 centimorgans (cM), with an average marker interval of 0.89 cM. High-resolution genetic maps are a useful tool for both marker-assisted breeding and various genome investigations in catfish, such as sequence assembly, gene localization, QTL detection and genome structure comparison. Hence, such a high-density linkage map will serve as a valuable resource for comparative genomics and fine-scale QTL mapping in catfish species.

## 1. Introduction

Discovering the genes or chromosomal regions that control morphological, physiological and behavioural characteristics is critical for understanding adaptive evolution and the evolutionary responses of natural populations. To identify such genes, a fine linkage map, which is an ordered listing of genetic markers located along the chromosomes in the genome, is needed. The linkage map is obtained by calculating the recombination frequency between genetic markers. It shows the relative position between genetic markers rather than the physical location of the genes on the chromosome. The phenomenon of genetic linkage underpins the genetic map. The construction of the linkage map provides an essential basis for identifying chromosomal regions and quantitative trait loci (QTLs) by genetic linkage analysis [1]. Linkage maps also serve as a link between genomic information of model organisms and that of related non-model organisms through the comparison of their genomes to facilitate the discovery of candidate genes of non-model organisms [2–4].

Genetic markers form the core of modern genetics and are basic tools to study adaptive evolution, ecological genetics and population genetics. After the introduction of the restriction fragment length polymorphism (RFLP) technique [5], approaches using other molecular markers, such as random amplified polymorphic DNA (RAPD), amplification fragment length polymorphism (AFLP), simple sequence repeats (SSRs) and single-nucleotide polymorphisms (SNPs), have also been developed. In particular, SNPs have a greater advantage over the other marker types because single base-pair changes between individuals [6] are the source of the most abundant small-scale genetic variation. SNPs have become an increasingly important tool for molecular genetic analysis, such as linkage map construction in fishes (e.g. [7,8]) and other species (e.g. [9,10]). Because SNPs are widely used in genetic and genomic studies, particularly to identify and tag economically important traits, a method that can uncover hundreds of polymorphic markers spanning the entire genome in a single, simple and reliable experiment is of paramount importance.

The emergence of massively parallel next-generation sequencing (NGS) enables identification of a large number of genetic markers, construction of high-resolution linkage maps, and comparative genomics studies in both model and non-model species. Several methods have been developed for high-throughput genetic marker discovery and genotyping using restriction enzymes, including restriction site-associated DNA sequencing (RAD-seq) [11]. This technique allows exploration of SNPs adjacent to restriction enzyme sites by sequencing only the DNA-flanking sequences across the whole genome, with simultaneous genotyping of markers in multiple individuals [11–14]. RAD-seq has been successfully applied to various organisms for linkage map construction [15,16], population genetics [17], QTL analysis [16,18], linkage disequilibrium analysis [19], comparative genomics [20–22] and genome assembly [23].

Animal breeders and geneticists are continuously improving the methods of high-resolution linkage map construction to make them more rapid and cost-effective. A high-resolution linkage map is an excellent tool for the assembly of genome sequences, comparative analysis of synteny, dissection of complex genetic traits and molecular marker-assisted breeding. To date, researchers have constructed linkage maps in over 45 fish species [24]. However, the density of these maps constructed in fish, e.g. in bighead carp (*Aristichthys nobilis*) [25], bighead catfish (*Clarias macrocephalus*) [26], mirror carp (*Cyprinus carpio carpio*) [27], common carp (*Cyprinus carpio*) [28] or channel catfish (*Ictalurus punctatus*) [29,30], is still low. With the development of NGS, it has become easier to construct high-density SNP-based linkage maps for various economically important fish species, such as channel catfish (*Ictalurus punctatus*) [8,31], large yellow croaker (*Larimichthys crocea*) [32], rainbow trout (*Oncorhynchus mykiss*) [33], Nile tilapia (*Oreochromis niloticus*) [34], Japanese flounder (*Paralichthys olivaceus*) [35], Atlantic salmon (*Salmo salar*) [36] and yellowtail (*Seriola quinqueradiata*) [37]. For example, by using the linkage map of farmed Atlantic salmon (*Salmo salar*) with 6712 SNPs, 50 QTL-linked SNPs were found to be associated with the resistance to infectious pancreatic necrosis [38]. Thus, such high-density genetic maps have shown significant advantages in fine QTL mapping, comparative genomics and assembly of genome sequences.

Southern catfish (*Silurus meridionalis*), also known as Chinese large-mouth catfish, is the largest catfish in China. It is also one of the most widely cultivated catfish species for food consumption in China. Although it is an economically important fish, its genetic resources are very limited. To facilitate genetic and genomic studies in *S. meridionalis*, the development of its high-resolution genetic map is imperative. The aim of the present study was to construct a high-resolution genetic linkage map for *S. meridionalis* to facilitate QTL analysis, comparative genomics, genome assembly and selective breeding of this economically important fish species.

## 2. Material and methods

We declare that the sample locations were public, and no specific permission was required to access these locations. The studied fish species was not protected under any wildlife conservation programmes. Euthanasia methods followed the guidelines of the Journal of the American Veterinary Medical Association (AVMA guidelines) [39]. All experiments, especially the use of ethanol for euthanasia, were conducted in accordance with the regulations provided by the guide for care and use of laboratory animals and were approved by the Committee of Laboratory Animal Experimentation of the Southwest University (Chongqing, China).

### 2.1. Mapping population and DNA extraction

The full-sib F1 population consisted of 200 progeny from an intraspecific cross between two unrelated wild fish caught from the upper Yangtze River in 2012. The resource family was reared in 1000 litre tanks until the collection of tissue samples for genotyping. In total, 100 progeny were randomly sampled for genetic map construction. Fresh muscle and both pelvic and pectoral fins from each individual were removed and preserved in 95% ethanol at −20°C before DNA extraction. The remaining tissues of each fish were stored in 95% ethanol at room temperature and labelled with a unique voucher number. All of the DNA samples were extracted from small pieces of fins using a DNeasy Blood and Tissue kit (Qiagen, Shanghai, China). The residual RNA was removed from the genomic DNA (gDNA) by RNase treatment. The quality and quantity of gDNA were determined using a NanoDrop 2000 UV-vis spectrophotometer (Thermo Fisher Scientific Inc., Wilmington, DE, USA). The integrity of the DNA in every sample was evaluated by agarose gel electrophoresis.

### 2.2. Library construction and RAD sequencing

Approximately 0.3–0.5 µg of genomic DNA was digested in a 50 µl reaction volume for 60 min at 37°C with 20 units of *Eco*RI (New England Biolabs, Ipswich, MA, USA) that recognizes the 5′-G/AATTC3-3′ sequence. The RAD sequencing protocols applied were as described by Baird *et al*. [11], except for several modifications. To track different samples, we used a modified Illumina P1 adapter containing individual-specific nucleotide barcodes with a length of 4–8 bp. At least two nucleotides differed in all barcodes to minimize sample misassignment due to sequencing error. The adapter-ligated fragments included in different samples within each library were subsequently pooled and randomly sheared with a Covaris E210 device (Covaris, Woburn, MA, USA) to an average size of 450 bp. They were then size-selected by electrophoresis on a 1% agarose (Sigma, Shanghai, China) 0.5× TBE gel from 350 bp to 550 bp and isolated using a MinElute Gel Extraction Kit (Qiagen, Shanghai, China). A modified Illumina P2 adapter was ligated, following which double-stranded DNA ends were treated with end-blunting enzymes, and 3′-adenine overhangs were added. Finally, libraries were enriched by PCR amplification, and the PCR products were sequenced on an Illumina HiSeq 2000 using paired-end reads (90 bp).

### 2.3. RAD-seq data processing

The raw reads were processed by deleting the reads with adapter sequences and removing the reads containing more than 50% of low-quality bases (quality value, ≤ 5). Then, all reads were assigned to the individuals by unambiguous barcodes with one mismatch. Reads without the unique barcodes or specific sequence were discarded. Finally, 1548.19 million clean reads were further trimmed to the RAD tags with a uniform length of 82 nucleotides (nt). Each RAD tag comprised 5 nt of the *Eco*RI recognition site and 77 nt of the potentially variable sequence. To obtain the SNPs, Rainbow 2.02 [40] was used to assemble RAD paired-end data for the male, female and both parents (the lengths of the first and second reads were 82 bp and 90 bp, respectively). Default parameters were used except for the minimum number of reads used to generate a contig, which we chose to be 10. Contigs shorter than 200 bp from both parents were removed. SOAP2.02 [41] was used to map the parents' paired-end RAD reads onto the reference contigs. SNPs were detected by SOAPsnp [42] to calculate the likelihood of genotypes for each individual. SNPs with a sequencing depth above 200 were removed as RAD sequences that were too abundant and probably represented repetitive sequences in the genome [43]. The genotypes that differed between the two parents were treated as potential high-quality SNP markers if the following criteria were satisfied: sequencing depth of 5–200 and base quality of ≥25. For the linkage genetic map construction, Stacks version 0.9998 pipeline [44] was used, and the RAD data showed a substantial increase in the total number of parents' putative SNPs at the tails of the sequences (the last eight nucleotides from positions 75 to 82) (electronic supplementary material, additional file 1), suggestive of sequencing errors, as described in earlier reports [45,46]. These were consequently removed from the analyses. For subsequent analyses, the final read length was trimmed to 74 nucleotides, and only the first (left) paired read was used.

DNA fragments created by RAD-tag library preparation have a restriction site at one end and are randomly sheared at the other end, so that the second paired-end reads are less suitable for SNP calling because of lower coverage than that of the first reads [43]. The clean reads were then used to assemble the RAD sequences into loci. Alleles were identified, and SNPs were detected using Stacks version 0.9998. First, the RAD tags were clustered into exactly matching stacks (ustacks). A minimum stack depth of five was required to create a stack in parents and progeny; a maximum sequence mismatch of two was allowed between stacks to merge into a locus within an individual. Second, a catalogue of the possible parents' loci was created and three mismatches were allowed between male and female tags when generating the catalogue. Third, each individual was matched against the catalogue. Finally, at the genotyping stage, a minimum stack depth of 10 reads was used, which was the number of exactly matching reads that had to be found to create a stack in an individual while correcting for the heterozygous alleles neglected due to their low coverage depth.

### 2.4. Linkage map construction

Only loci that were polymorphic within one or both parents could be mapped according to the double pseudo-test cross strategy [47], and markers showing significantly distorted segregation (*p*-value < 0.01) were excluded from map construction. Linkage analysis was performed for at least 80% of the markers by using Lep-MAP [48]. The genotype data were first filtered manually to remove obvious Mendelian errors from the offspring. Linkage groups (LGs) were identified with a logarithm of odds score limit of 10. The marker order was obtained using the OrderMarkers module of Lep-MAP [48]. The eventual linkage maps were drawn using MapChart v. 2.2 [49].

### 2.5. Sequence comparison

Consensus sequences of RAD-tag markers (75 bases in length) were aligned with chromosome sequences of channel catfish (*Ictalurus punctatus*). The channel catfish genome sequences were retrieved from ftp://ftp.ncbi.nlm.nih.gov/genomes/all/GCA_001660625.1_IpCoco_1.2/GCA_001660625.1_IpCoco_1.2_assembly_structure/Primary_Assembly/assembled_chromosomes/FASTA/, and compared by the method previously described by Kakioka *et al*. [50]. In brief, The RAD-tag markers were mapped to the channel catfish genome sequences using the BLAST software suite (NCBI BLAST+ version 2.2.26) with an *e*-value cut-off of 10^−10^. In cases where the search of a query sequence hit two or more loci, the hit with the smallest *e*-value was considered significant, whereas if the difference in *e*-values between the first and the second smallest hits was not greater than 10^3^, the hit was considered insignificant [50].

## 3. Results and discussion

### 3.1. RAD sequencing and SNP discovery

Taking advantage of massively parallel sequencers, we constructed and characterized a high-density SNP linkage map of southern catfish derived from RAD-seq. The genomic DNA of each sample was digested using the restriction enzyme *Eco*RI. Nine RAD libraries from the F1 population (two parents and their 100 progeny) were constructed and sequenced.

Approximately 145.02 G bases (approx. 1611 M reads) were generated (raw data) for all pooling lanes. After quality filtering, 141.99 G bases were generated (clean data) and retained. All libraries were split into different samples by barcoding, yielding 139.34 G (approx. 1548 M reads) bases for the F1 population. As shown in the electronic supplementary material, additional file 2, 16.99 M reads and 15.14 M reads, on average, were produced for parents and progeny, respectively. The Q20 value (*Q* ≥ 20) of each sample was above 98%; therefore, the quality of the data was very high. According to Zhao *et al*. [46], the parents' RAD paired-end reads can be useful for assembly into consensus contig sequences and discovery of SNPs. Based on the methods of locally assembling RAD tags, some software packages have been developed to identify SNPs and define putative haplotypes in populations [14,40,51]. Here, the two parents' RAD data were assembled into 241 007 contigs with a mean length of 246 bp (N90 = 246 bp) (table 1) using the software Rainbow 2.0 [40], which suggests that most of the SNPs can be used as candidate markers for marker-assisted selection or breeding. The distribution of contig lengths, as shown in the electronic supplementary material, additional file 3, had two peaks. The total contig lengths for male, female and both were 68.89 Mb, 60.15 Mb and 77.83 Mb, respectively, which is approximately 10% of the southern catfish genome (the total size of the catfish genome is 780 Mb according to *k*-mer analysis; unpublished data).

**Table 1.** RAD paired-end contig assembly.

	male		female		male and female	
	contig length (bp)	number	contig length (bp)	number	contig length (bp)	number
N50	278	220 045	267	200 185	286	125 758
N60	271	192 354	260	174 730	280	153 274
N70	264	165 855	252	150 438	272	181 454
N80	255	140 143	243	126 953	263	210 510
N90	241	115 049	229	104 113	246	241 007
total_length	68 892 708		60 150 047		77 828 008	
maximum length	699		1136		782	
number ≥ 200 bp		250 708		227 972		275 278
GC_rate		0.382		0.381		0.382

**Figure 1.**
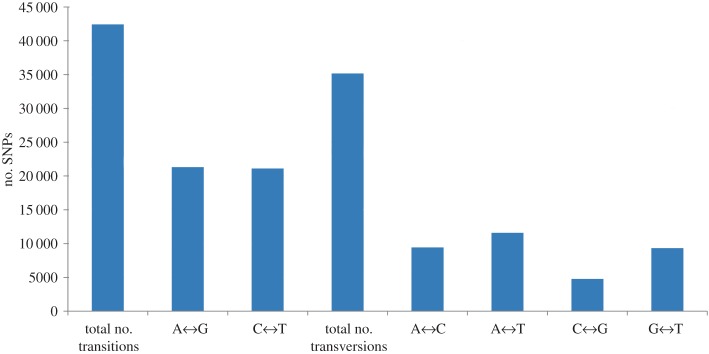
Transitions and transversions within 77 634 bi-allelic single-nucleotide polymorphisms (SNPs) detected among southern catfish parents.

Between the parents, 77 634 high-quality SNPs with a sequencing depth of ≥5× and base quality of ≥25 were detected (electronic supplementary material, additional file 4). Nucleotide variations of 77 634 SNPs in southern catfish were bi-allelic, of which 54.7% were transitions and 45.3% were transversions (figure 1). Thus, the rate of transitions was higher (transition/transversion ratio of 1.20). In brief, 42 447 transitions (A/G and C/T) and 35 187 transversions (A/C, A/T, C/G and G/T) were detected among polymorphic SNPs, with A/G being the most common (50.3%) and C/G being the least common (13.6%) substitutions observed, which was similar to the findings reported in other vertebrate species [52].

### 3.2. Stacks analysis in F1 population

The Stacks software is a useful analysis tool set for population genomics [44,46,53]. More than 6.16 million RAD-tag sequences were generated in each of the two parents using the pipeline of Stacks version 0.9998. These sequences were clustered and counted, which yielded more than 270 000 unique stacks in each parent (electronic supplementary material, additional file 5). A set of 304 782 stacks and 247 310 shared stacks contained both monomorphic and polymorphic RAD tags. The 304 782 stacks were the total stacks of the combination of male and female unique stacks (merged the shared stacks). In total, 62 547 and 24 880 putative SNPs were found in the female and male parent, respectively, and 56 987 RAD markers between the parents were screened (table 2). With respect to the RAD-tag sequences, the heterozygosity rate in Pfemale (maternal parent) (0.308%) was much higher than that in Pmale (paternal parent) (0.121%; table 2).

**Table 2.** RAD-tag and SNP discovery in the parents. Note: Filtered RAD tags are the number of the reads with the specific recognition site (AATTC). The unique stacks are the number of clustered RAD-tag sequences found in the data from each parent using ustacks with two mismatches. The shared stacks are the number of unique stacks shared between the two parents including both polymorphic and monomorphic loci. Putative SNPs are the stacks containing loci in each parent. The heterozygosity rate is calculated as the ratio of the number of SNPs to the total length of the unique stacks in each parent. The RAD markers are the different loci between the two parents by matching the loci from each parent against the catalogue with a 10-fold minimum stack depth.

parent	filtered RAD tags	unique stacks	shared stacks	putative SNPs	heterozygosity rate	RAD markers
Pfemale	6 162 903	274 293	247 310	62 547	0.308%	56 987
Pmale	9 958 522	277 799	247 310	24 880	0.121%	56 987

### 3.3. Linkage map construction

To date, there have been several attempts to construct catfish genetic maps, but most of them were in channel catfish (*Ictalurus punctatus*) [8,29,30]. No high-density linkage maps for any silurid catfish species have been obtained. In the present study, a high-resolution RAD-based SNP genetic map of *S. meridionalis* was constructed using Lep-MAP for the first time. The integrated linkage map consisted of 29 LGs comprising 26 714 SNPs (table 3), which corresponded to 6715 effective loci. The effective loci have different genetic distances, i.e. SNPs that have the same genetic distances represent only one effective locus. The number of LGs was congruent with the number of chromosomes of both southern catfish (2*n* = 58) [54,55] and *Silurus asotus* (2*n* = 58) [56], a species belonging to the same genus as southern catfish. The total integrated map length was 5918.31 cM, with a mean distance between markers of 0.89 cM. Moreover, the maximal interval between genetic RAD loci was 49.27 cM (LG6), whereas the minimal one was 0.01 cM. The genetic length of each LG ranged from 62.59 cM (LG29) to 445.84 cM (LG4), with an average interval between loci of 0.44–1.77 cM. Moreover, the number of markers mapped on an LG varied from 125 (LG29) to 331 (LG3) per group. LG24 was the densest, having 235 effective loci with an average density of 0.44 cM, whereas LG29 had the lowest number of effective loci (only 125). On average, each LG contained 231.6 effective loci spanning 204.08 cM (table 3). Locus names and SNP positions on the 29 LGs of the genetic map are listed in the electronic supplementary material, additional file 6.

**Table 3.** A summary of the genetic linkage map constructed in southern catfish (*Silurus meridionalis*).

	maternal map		paternal map		integrated_map			
group	number of marker	genetic distance (cM)	number of marker	genetic distance (cM)	number of marker	genetic distance (cM)	no. of effective loci	average interlocus distance (cM)
LG1	1269	253.80	243	218.97	1424	303.83	287	1.06
LG2	1144	365.98	209	167.24	1276	389.37	289	1.35
LG3	1121	285.71	227	462.22	1264	337.69	331	1.02
LG4	1049	521.91	208	367.79	1195	445.84	288	1.55
LG5	1031	182.77	172	268.29	1149	252.78	343	0.74
LG6	1027	593.78	199	294.68	1147	417.85	236	1.78
LG7	971	257.45	173	218.48	1103	283.41	245	1.16
LG8	949	141.25	185	155.80	1059	179.72	275	0.66
LG9	894	163.47	156	148.03	991	148.89	195	0.77
LG10	901	106.97	137	102.95	987	105.11	208	0.51
LG11	868	177.06	133	180.61	965	191.38	242	0.79
LG12	858	110.41	160	131.55	961	134.48	239	0.57
LG13	819	145.58	180	282.80	942	233.04	271	0.86
LG14	836	120.21	157	252.61	942	221.64	206	1.08
LG15	825	115.72	153	113.76	931	172.90	222	0.78
LG16	806	113.77	151	142.95	911	144.19	246	0.59
LG17	765	202.95	163	130.17	890	244.45	258	0.95
LG18	793	234.10	150	200.00	890	217.55	176	1.24
LG19	704	269.80	136	157.02	786	204.76	217	0.95
LG20	675	91.81	140	144.38	771	131.38	262	0.50
LG21	680	236.81	127	161.83	770	236.22	190	1.25
LG22	682	88.44	126	94.95	762	90.38	195	0.47
LG23	644	242.23	149	237.80	743	249.95	233	1.08
LG24	645	77.99	129	98.10	738	102.49	235	0.44
LG25	613	84.70	131	86.30	697	108.96	179	0.61
LG26	599	86.31	131	100.78	686	111.20	171	0.65
LG27	592	99.87	129	95.62	669	117.13	195	0.60
LG28	550	68.39	87	71.26	603	79.13	156	0.51
LG29	418	63.15	73	58.45	462	62.59	125	0.50
total:	23 728	5502.39	4514	5145.39	26 714	5918.31	6715	0.89

Currently, most catfish genetic maps are constructed using expressed sequence tag (EST) [57], AFLP [26,29,58] and SSR [30,57,58]. The density of these linkage maps in catfish is still low, with an average density of 1.40–15.20 cM. The average density of the southern catfish linkage map in this study was much higher than those described above. Furthermore, a fine linkage map is an essential tool for genomics. A high-density linkage map, such as the one we constructed, provides detailed information on genomic structure and is a valuable resource for comparative genomics and fine-scale QTL mapping in catfishes.

### 3.4. Analysis of recombination rates

**Figure 2.**
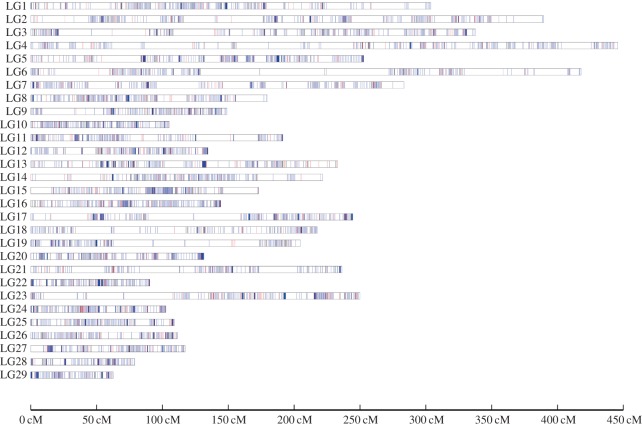
The linkage map of southern catfish (*Silurus meridionalis*). Within each linkage group, red, blue and black lines, respectively, represent maternal heterozygous SNPs, paternal heterozygous SNPs and SNPs heterozygous in both parents. Genetic map details are given in the electronic supplementary material, additional file 7.

Recombination refers to the occurrence of gene combinations in the progeny that differ from those of the parents as a result of independent assortment and crossing over. It plays a central role in genetics. Within each LG (figure 2 and electronic supplementary material, additional file 7), mild-to-strong localized, specific recombination patterns were observed. Strongly labelled regions may be regarded as recombination hotspots, whereas weakly labelled regions may indicate no recombination or missing markers. Large intervals were often found in some LGs, possibly because no recombination events occurred during meiosis between the markers used for mapping analysis. In our study, large intervals were found in LG6, indicating that some regions of the southern catfish genome had extremely low recombination rates. This suggests the presence of a centromere or repetitive region in this LG, given the higher recombination rate near the telomeres and lower recombination rate near the centre of the chromosomes [59]. It has, however, also been observed that genetic recombination rates might be artificially inflated by genotyping errors or if markers with large amounts of missing data are included in the analysis (e.g. [60]).

### 3.5. Syntenic relationship between southern catfish and channel catfish

**Figure 3.**
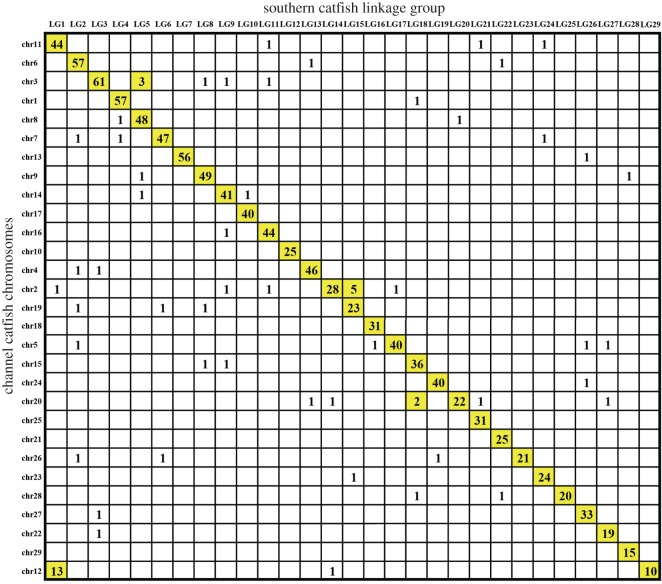
Comparison of 29 linkage groups of southern catfish with the genome of channel catfish (*Ictalurus punctatus*; 29 chromosomes).

BLAST searches of the mapped southern catfish RAD-tag marker consensus sequences against the genome sequences of channel catfish indicated a syntenic relationship between them. Generally, the organization of the southern catfish genome is similar to that of channel catfish such that synteny can be identified. Of the hits against the channel catfish genome, most were aligned to the chromosomes that had a one-to-one relationship with southern catfish linkage groups. Karyotypes of southern catfish and channel catfish both have 29 chromosomes (2*n* = 58) [31,55], 27 (93.10%) of which have a simple one-to-one relationship. For example, nearly all of the southern catfish RAD-tag marker consensus sequences in southern catfish LG2 were in channel catfish chromosome (chr) 6. This suggests highly conserved synteny between southern catfish and channel catfish (figure 3).

Most of the RAD loci located in southern catfish LGs had a good syntenic relationship with a single channel catfish chromosome, and most of the LGs had one-to-one relationships with channel catfish chromosomes, except that LG1 corresponded to chr11 and chr12, whereas chr12 shared loci with LG1 and LG29 (figure 3). This suggested that southern catfish and channel catfish have high chromosomal collinearity, as they belong to the same order Siluriformes. The result is consistent with previous comparative analyses, which indicated that inter-chromosomal rearrangements are less frequent in teleost fishes than in mammals [61–65]. Although synteny was observed, chromosome fusion and fission are not rare. It is likely that extensive inter-chromosomal rearrangements have not occurred in either of the lineages that led to southern catfish and channel catfish since they diverged. However, our results also revealed one channel catfish chromosome (chr12) that shared loci with two southern catfish linkage groups (LG1 and LG29), whereas LG1 shared loci with chr11 and chr12. This could have resulted from a chromosome recombination or transposition followed by fusion/fission events [66].

Our results set the foundation for future analysis of the genetic mechanisms and evolutionary history that underlie the phenotypic diversity of catfishes, which are globally distributed and inhabit rivers, lakes, ponds, bog, paddy fields, channels and even underground water. Given these complex and different habitats, catfishes may be highly adaptable. However, evolutionary ecological studies of catfishes are rare. Comparative analyses can be used to investigate catfish genomes. Such studies may be used to predict candidate genes responsible for the traits that have ecological and evolutionary significance and are related to phenotypic divergence.

## 4. Conclusion

In the present study, we constructed a highly dense genetic linkage map of southern catfish and applied RAD sequencing in silurid catfish for the first time. The extensive synteny between southern catfish and channel catfish has been demonstrated, and differences between these species have also been identified. Our results establish the basis for the dissection of complex genetic traits and molecular marker-assisted breeding in catfish species. This map provides detailed genome sequence data for future gene-finding and genomic studies in this economically important catfish species. The map will also be a valuable resource for improving and validating silurid catfish whole-genome sequencing and assembly, which are currently underway.

## Supplementary Material

Additional file 1

## Supplementary Material

Additional file 2

## Supplementary Material

Additional file 3

## Supplementary Material

Additional file 4

## Supplementary Material

Additional file 5

## Supplementary Material

Additional file 6

## Supplementary Material

Additional file 6

## Data accessibility

The RAD sequencing raw data are available under accession numbers (SRP118891) at the NCBI Sequence Read Archive database (http://www.ncbi.nlm.nih.gov/Traces/sra). Additional results are available as electronic supplementary material, additional files 1–7.
