# Supplementary figures and images for: Restriction site-associated DNA sequencing for SNP discovery and high-density genetic map construction in southern catfish (Silurus meridionalis)
Source: R Soc Open Sci. 2018 May 30;5(5):172054. doi: 10.1098/rsos.172054 (PMC5990832; doi:10.1098/rsos.172054)

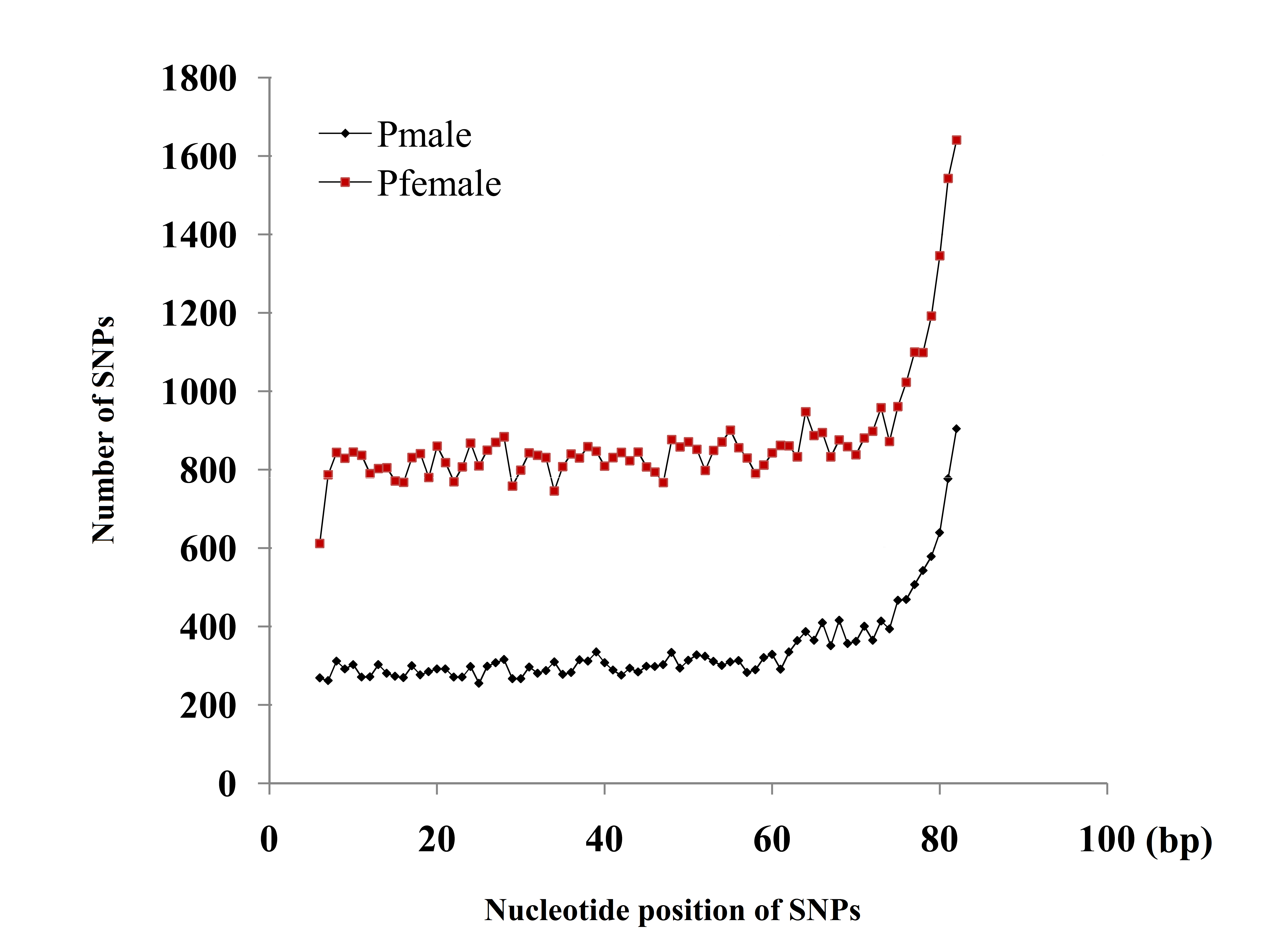

Supplement: Additional file 1 [file rsos172054supp1.tif]

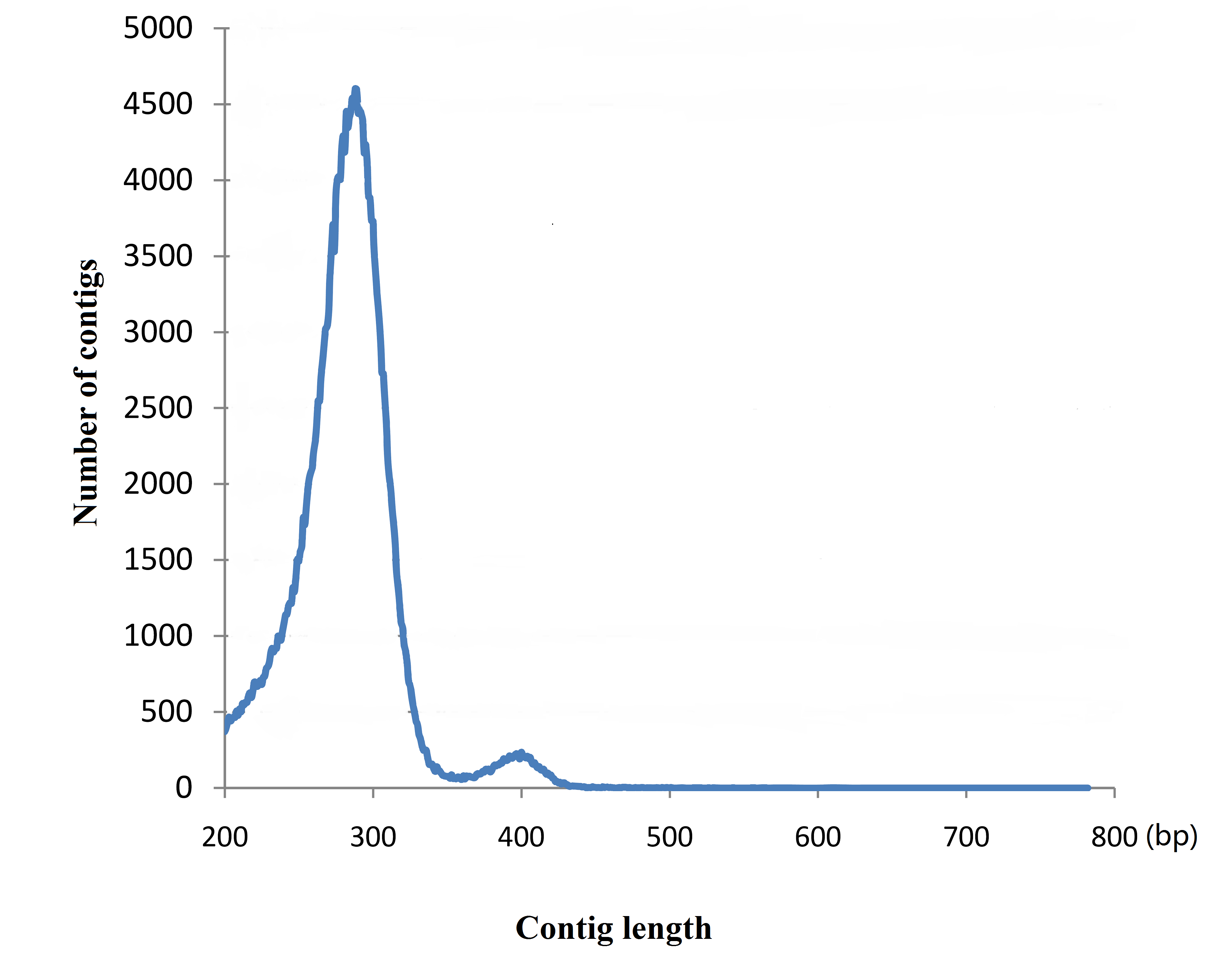

Supplement: Additional file 3 [file rsos172054supp3.tif]
