# Supplementary material for: Restriction site-associated DNA sequencing for SNP discovery and high-density genetic map construction in southern catfish (Silurus meridionalis)
Source: R Soc Open Sci. 2018 May 30;5(5):172054. doi: 10.1098/rsos.172054 (PMC5990832; doi:10.1098/rsos.172054)

Catfish Genetic Map

1 [1]

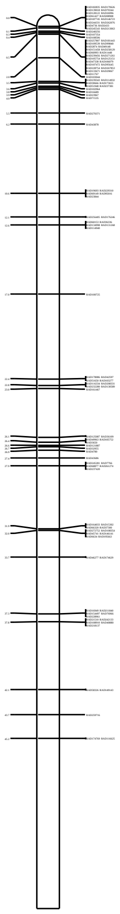

Catfish Genetic Map

1 [2]

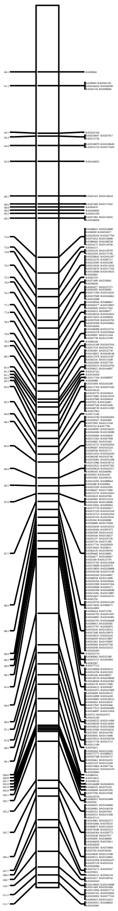

Catfish Genetic Map

1 [3]

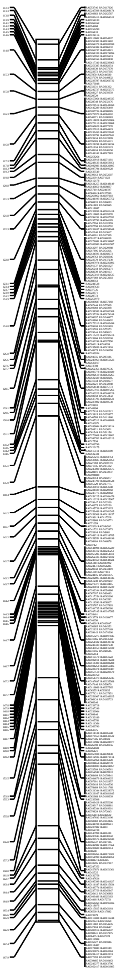

Catfish Genetic Map

1 [4]

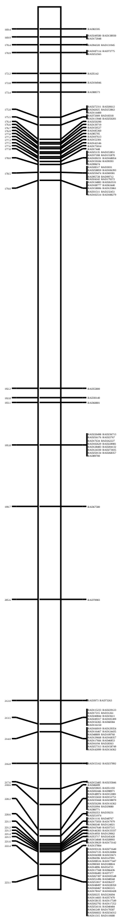

Catfish Genetic Map

1 [5]

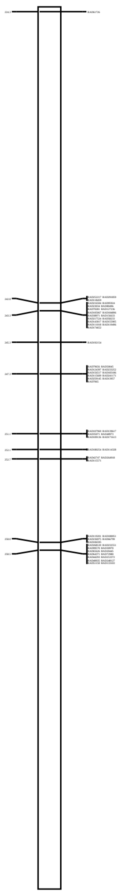

Catfish Genetic Map

1 [6]

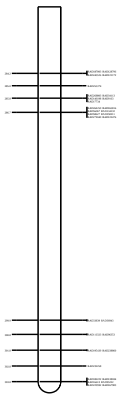

Catfish Genetic Map

2 [1]

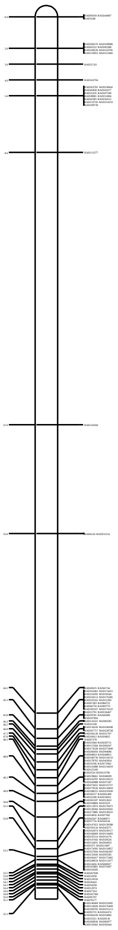

Catfish Genetic Map

2 [2]

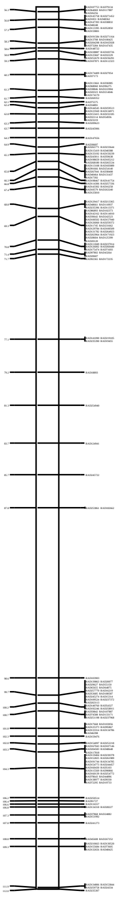

Catfish Genetic Map

2 [3]

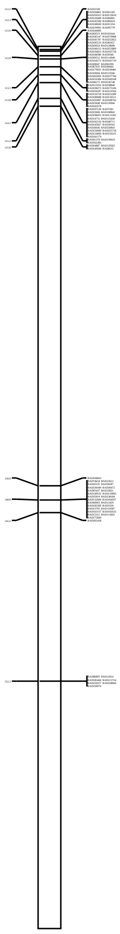

Catfish Genetic Map

2 [4]

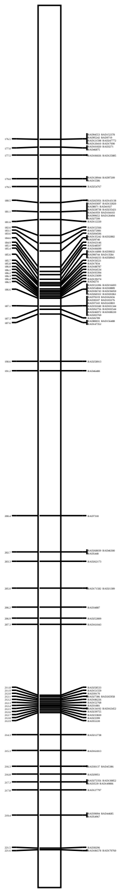

Catfish Genetic Map

2 [5]

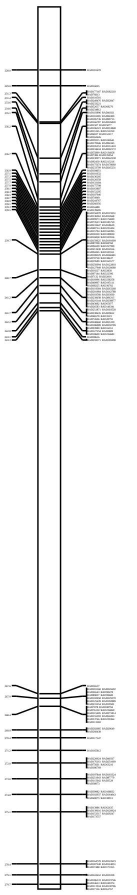

Catfish Genetic Map

2 [6]

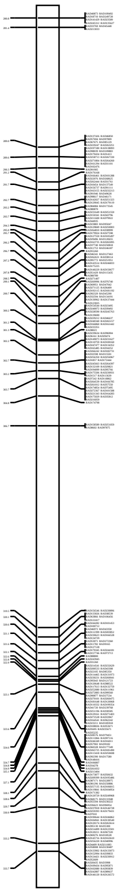

Catfish Genetic Map

2 [7]

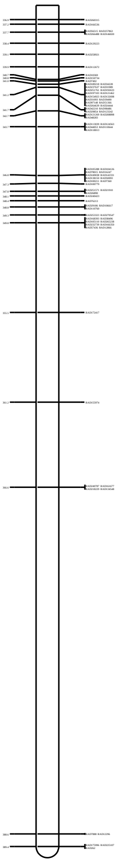

Catfish Genetic Map

3 [1]

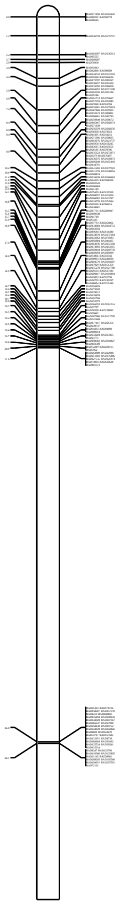

Catfish Genetic Map

3 [2]

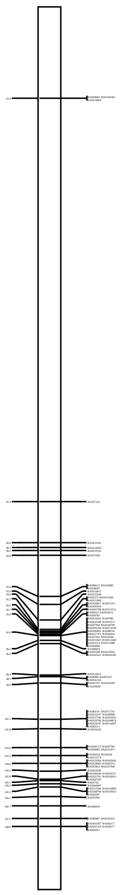

Catfish Genetic Map

3 [3]

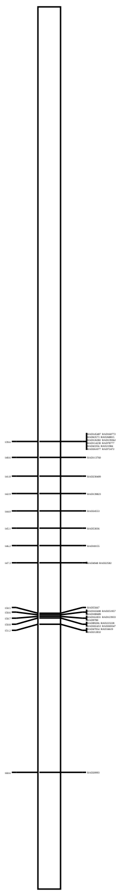

Catfish Genetic Map

3 [4]

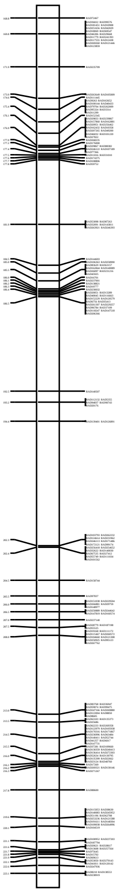

Catfish Genetic Map

3 [5]

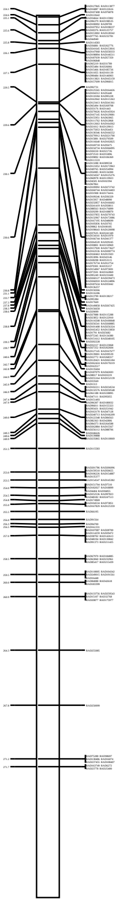

Catfish Genetic Map

3 [6]

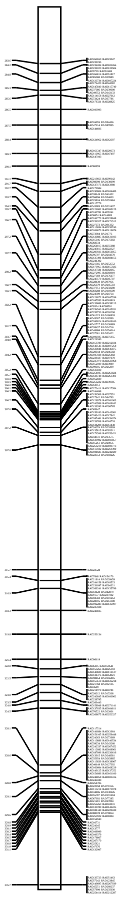

Catfish Genetic Map

3 [7]

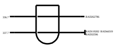

Catfish Genetic Map

4 [1]

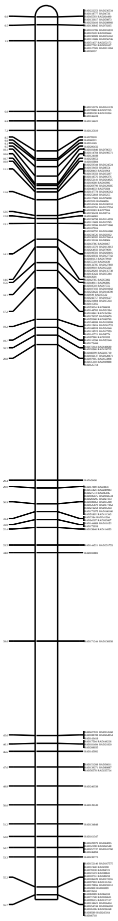

Catfish Genetic Map

4 [2]

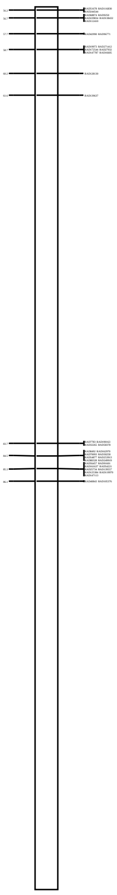

Catfish Genetic Map

4 [3]

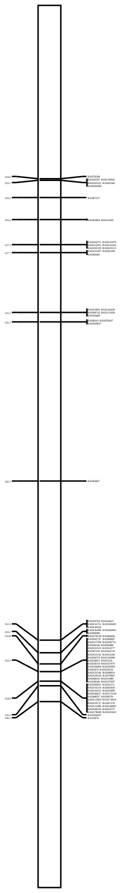

Catfish Genetic Map

4 [4]

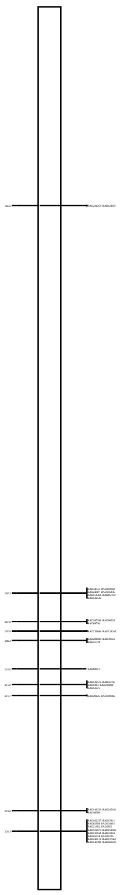

Catfish Genetic Map

4 [5]

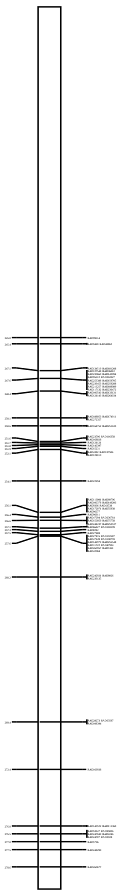

Catfish Genetic Map

4 [6]

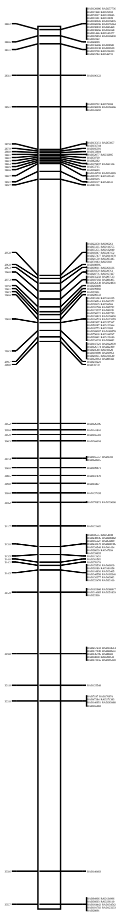

Catfish Genetic Map

4 [7]

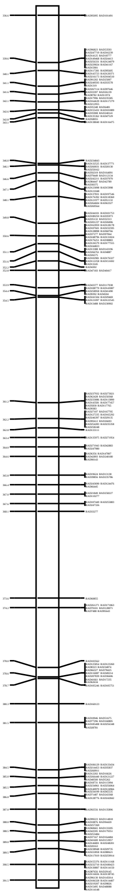

Catfish Genetic Map

4 [8]

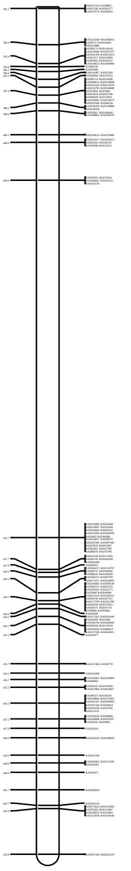

Catfish Genetic Map

5 [1]

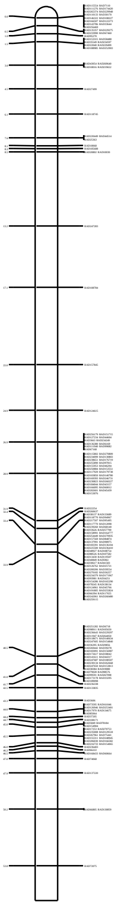

Catfish Genetic Map

5 [2]

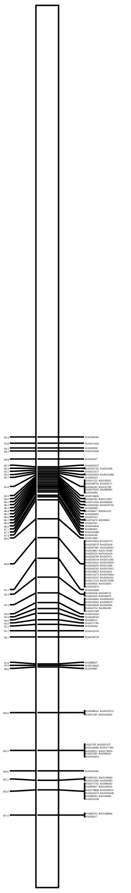

Catfish Genetic Map

5 [3]

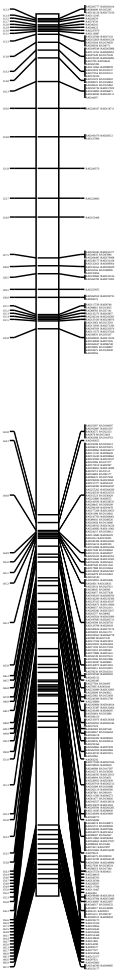

Catfish Genetic Map

5 [4]

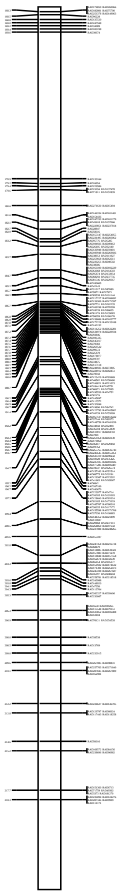

Catfish Genetic Map

5 [5]

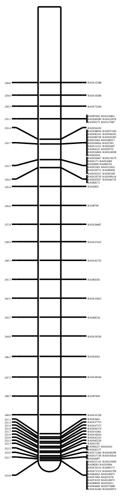

Catfish Genetic Map

6 [1]

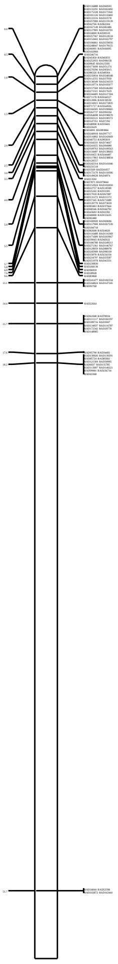

Catfish Genetic Map

6 [2]

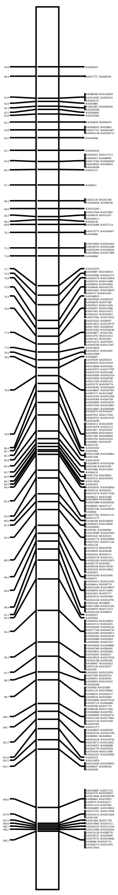

Catfish Genetic Map

6 [3]

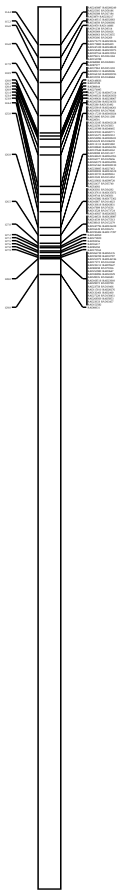

Catfish Genetic Map

6 [4]

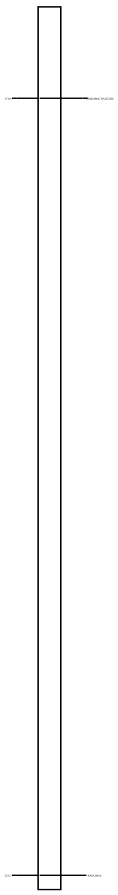

Catfish Genetic Map

6 [5]

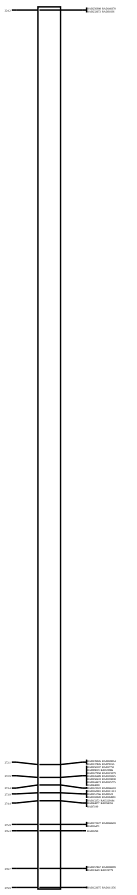

Catfish Genetic Map

6 [6]

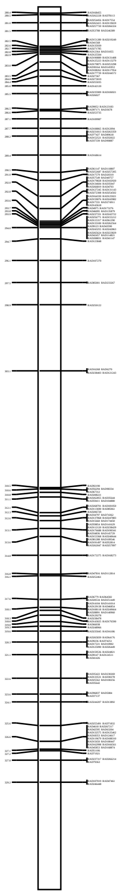

Catfish Genetic Map

6 [7]

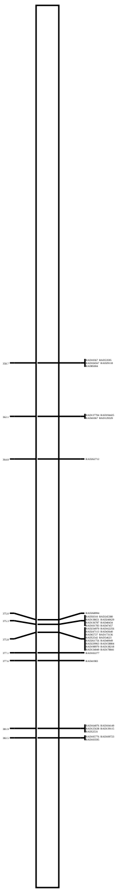

Catfish Genetic Map

6 [8]

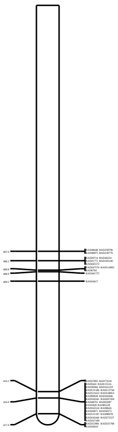

Catfish Genetic Map

7 [1]

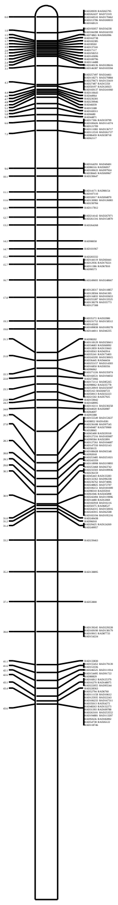

Catfish Genetic Map

7 [2]

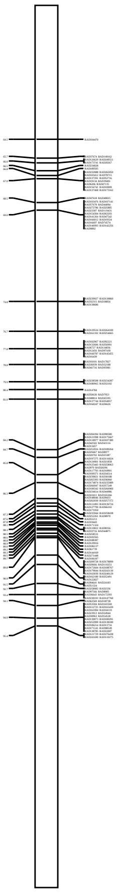

Catfish Genetic Map

7 [3]

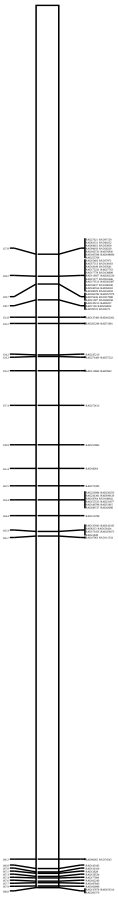

Catfish Genetic Map

7 [4]

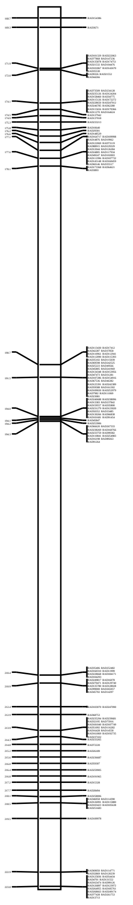

Catfish Genetic Map

7 [5]

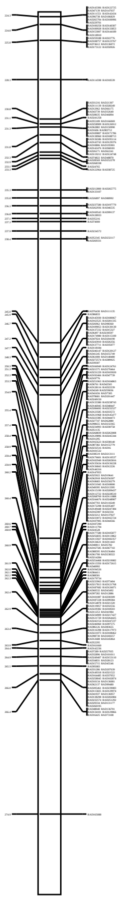

Catfish Genetic Map

7 [6]

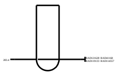

Catfish Genetic Map

8 [1]

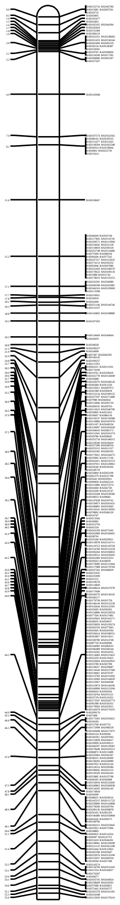

Catfish Genetic Map

8 [2]

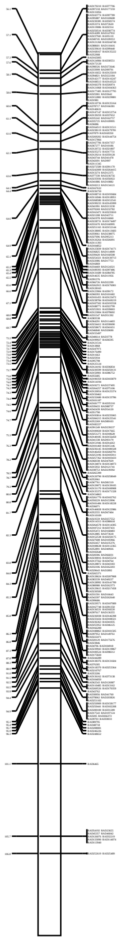

Catfish Genetic Map

8 [3]

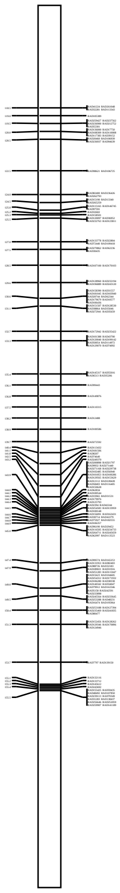

Catfish Genetic Map

8 [4]

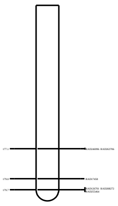

Catfish Genetic Map

9 [1]

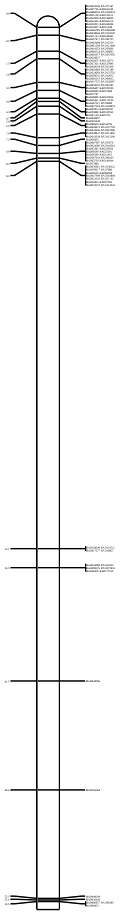

Catfish Genetic Map

9 [2]

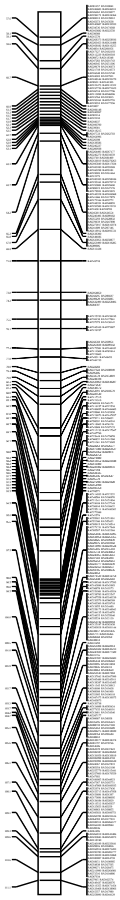

Catfish Genetic Map

9 [3]

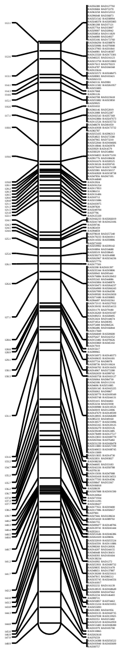

Catfish Genetic Map

10 [1]

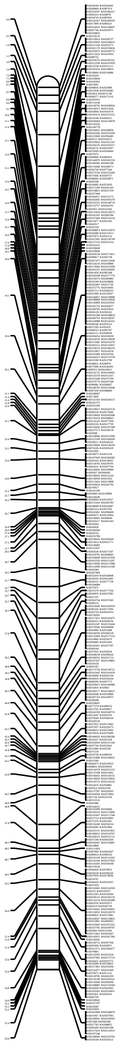

Catfish Genetic Map

10 [2]

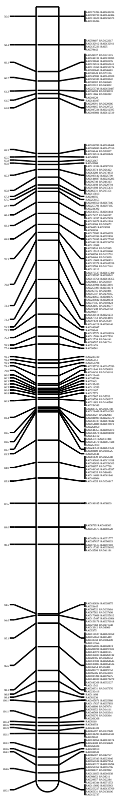

Catfish Genetic Map

11 [1]

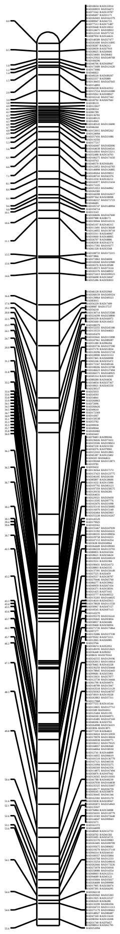

Catfish Genetic Map

11 [2]

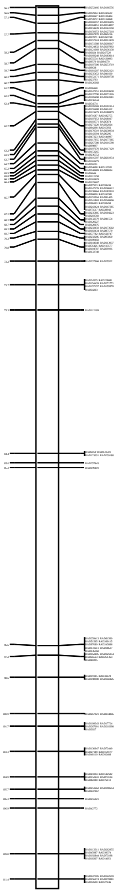

Catfish Genetic Map

11 [3]

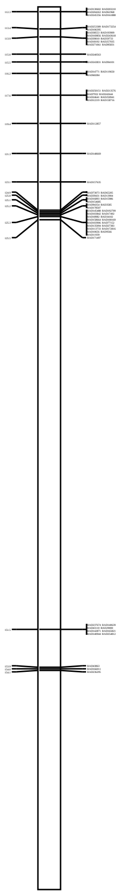

Catfish Genetic Map

11 [4]

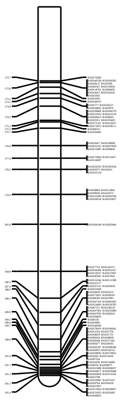

Catfish Genetic Map

12 [1]

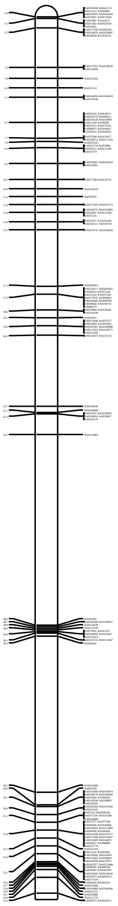

Catfish Genetic Map

12 [2]

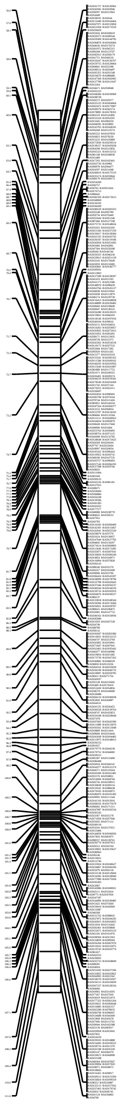

Catfish Genetic Map

12 [3]

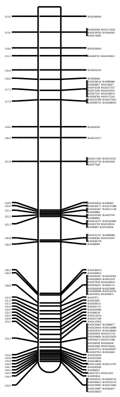

Catfish Genetic Map

13 [1]

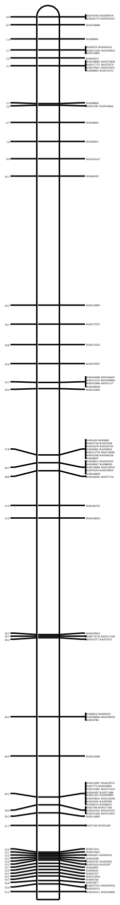

Catfish Genetic Map

13 [2]

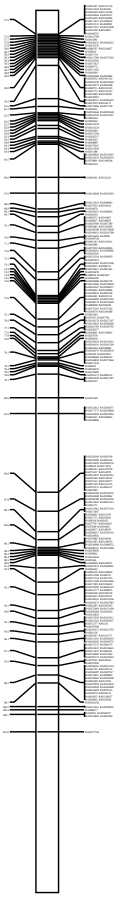

Catfish Genetic Map

13 [3]

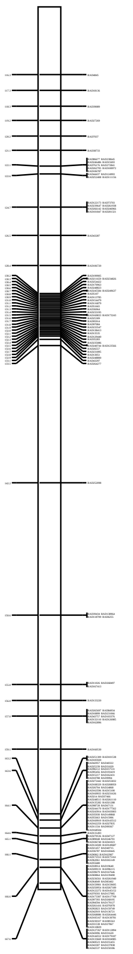

Catfish Genetic Map

13 [4]

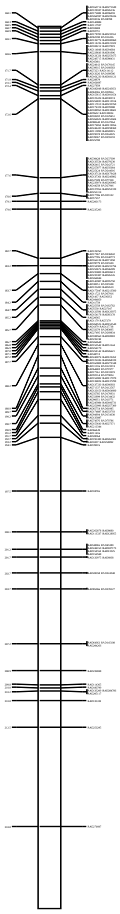

Catfish Genetic Map

13 [5]

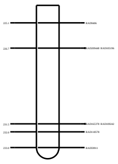

Catfish Genetic Map

14 [1]

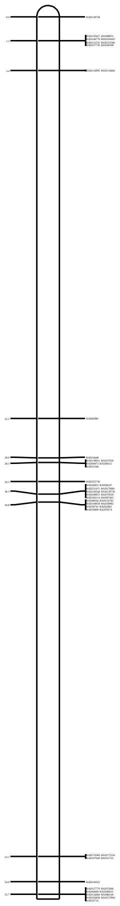

Catfish Genetic Map

14 [2]

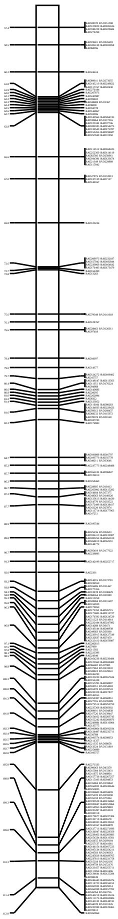

Catfish Genetic Map

14 [3]

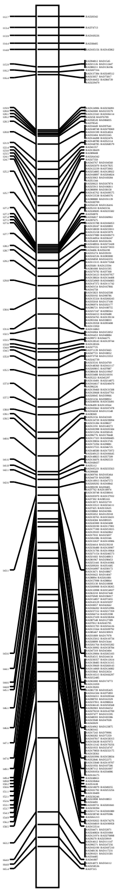

Catfish Genetic Map

14 [4]

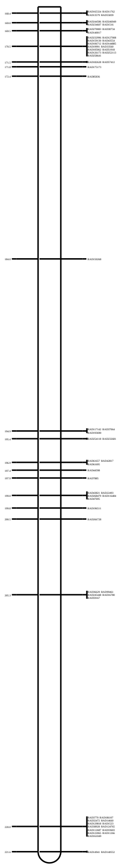

Catfish Genetic Map

15 [1]

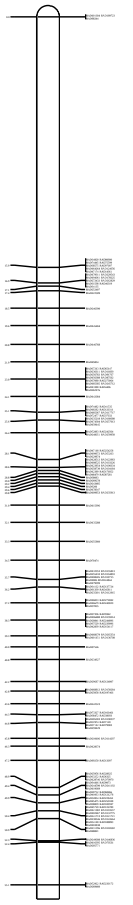

Catfish Genetic Map

15 [2]

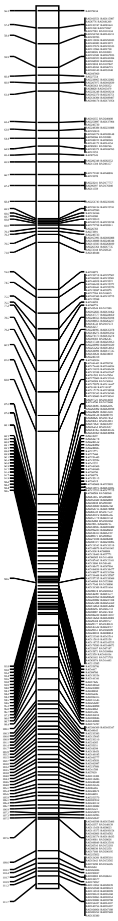

Catfish Genetic Map

15 [3]

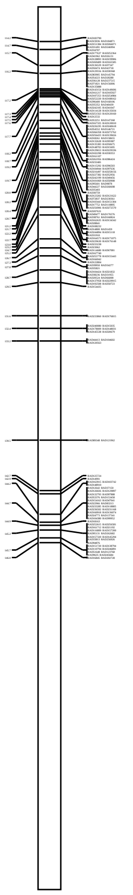

Catfish Genetic Map

15 [4]

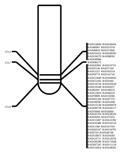

Catfish Genetic Map

16 [1]

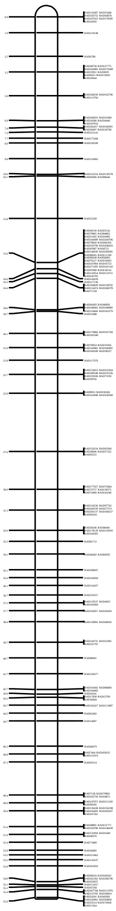

Catfish Genetic Map

16 [2]

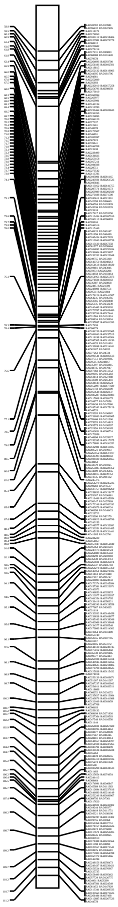

Catfish Genetic Map

16 [3]

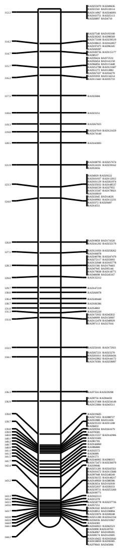

Catfish Genetic Map

17 [1]

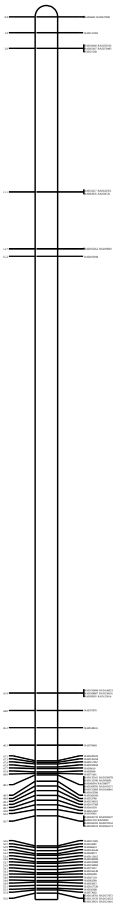

Catfish Genetic Map

17 [2]

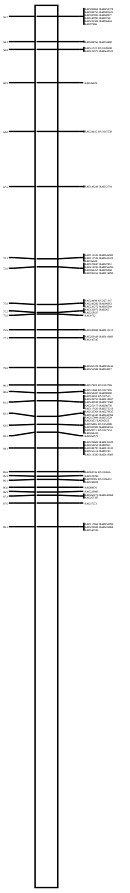

Catfish Genetic Map

17 [3]

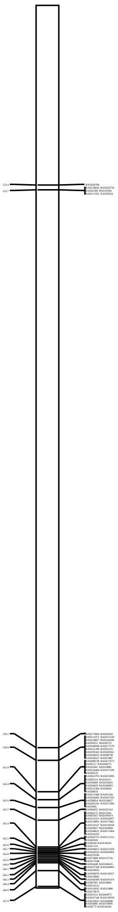

Catfish Genetic Map

17 [4]

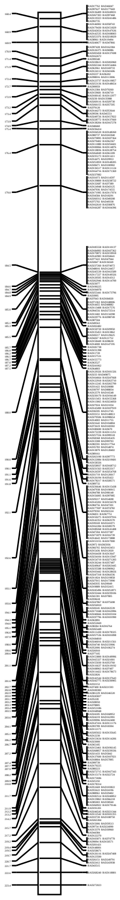

Catfish Genetic Map

17 [5]

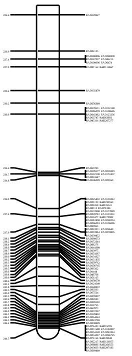

Catfish Genetic Map

18 [1]

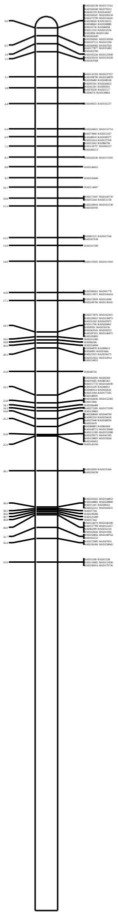

Catfish Genetic Map

18 [2]

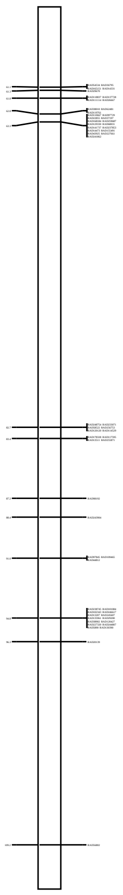

Catfish Genetic Map

18 [3]

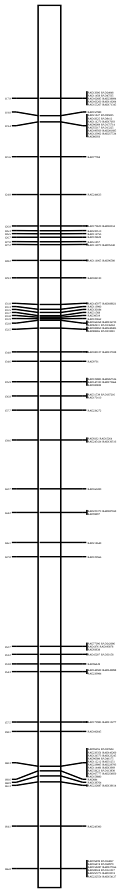

Catfish Genetic Map

18 [4]

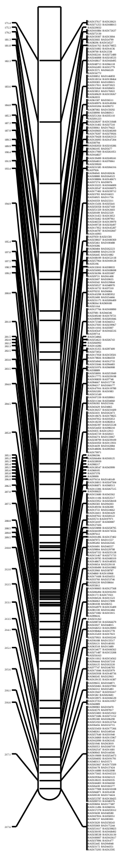

Catfish Genetic Map

19 [1]

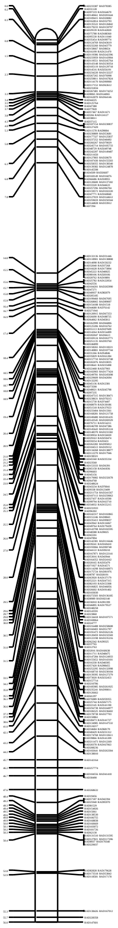

Catfish Genetic Map

19 [2]

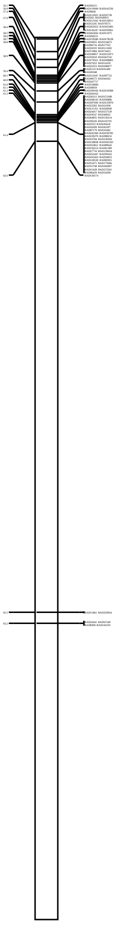

Catfish Genetic Map

19 [3]

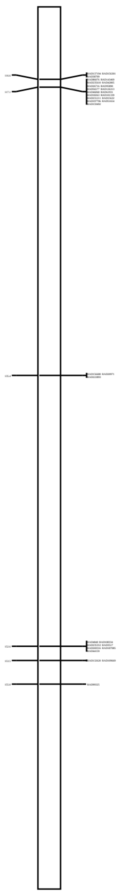

Catfish Genetic Map

19 [4]

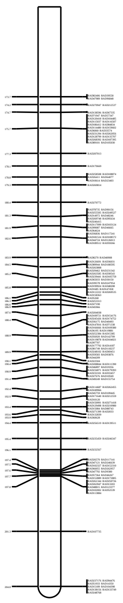

Catfish Genetic Map

20 [1]

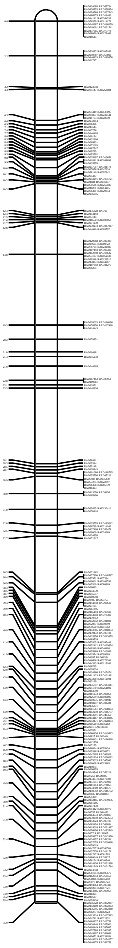

Catfish Genetic Map

20 [2]

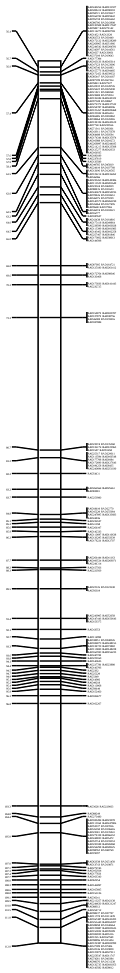

Catfish Genetic Map

20 [3]

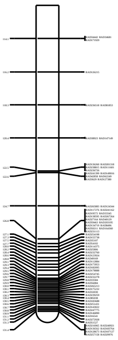

Catfish Genetic Map

21 [1]

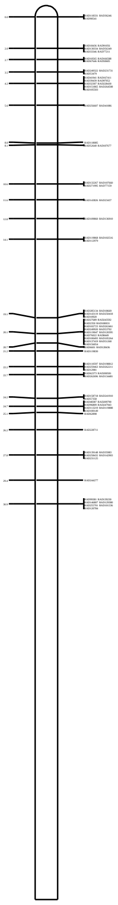

Catfish Genetic Map

21 [2]

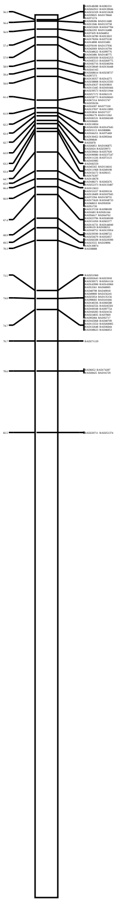

Catfish Genetic Map

21 [3]

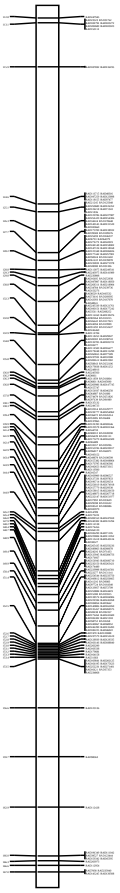

Catfish Genetic Map

21 [4]

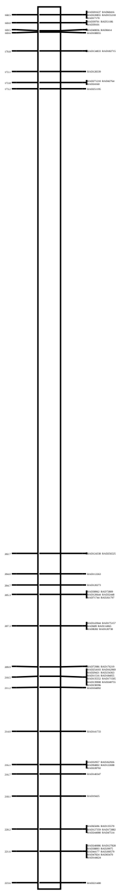

Catfish Genetic Map

21 [5]

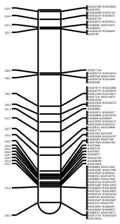

Catfish Genetic Map

22 [1]

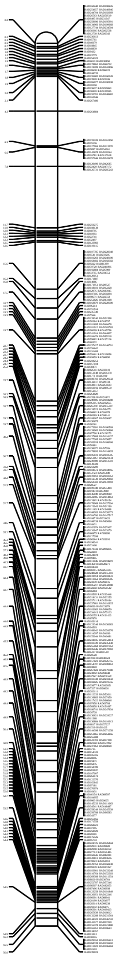

Catfish Genetic Map

22 [2]

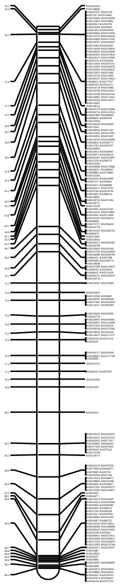

Catfish Genetic Map

23 [1]

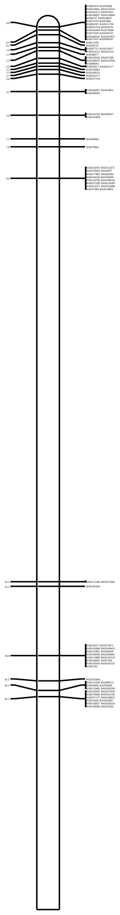

Catfish Genetic Map

23 [2]

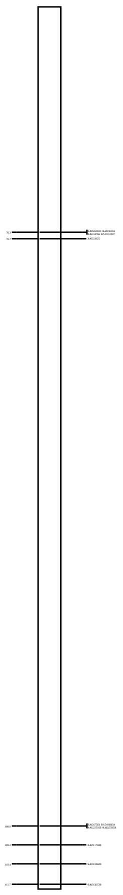

Catfish Genetic Map

23 [3]

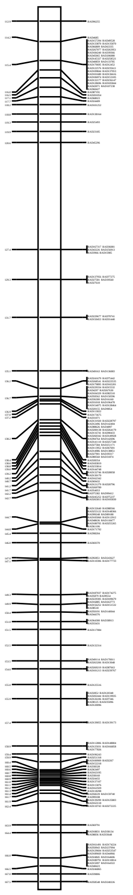

Catfish Genetic Map

23 [4]

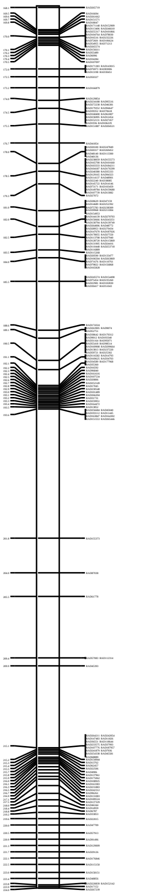

Catfish Genetic Map

23 [5]

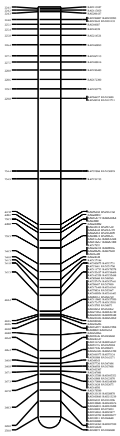

Catfish Genetic Map

24 [1]

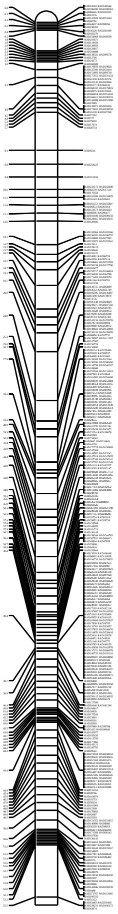

Catfish Genetic Map

24 [2]

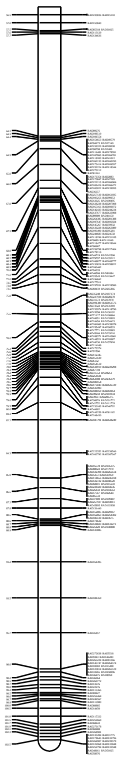

Catfish Genetic Map

25 [1]

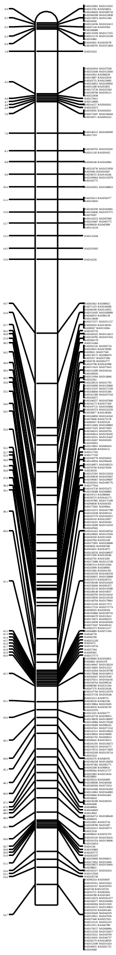

Catfish Genetic Map

25 [2]

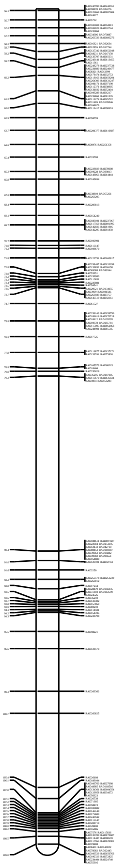

Catfish Genetic Map

26 [1]

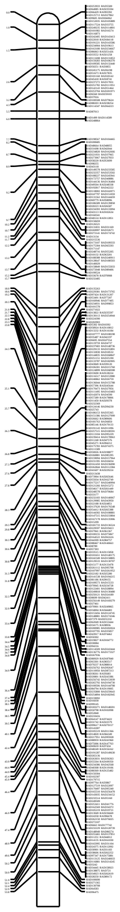

Catfish Genetic Map

26 [2]

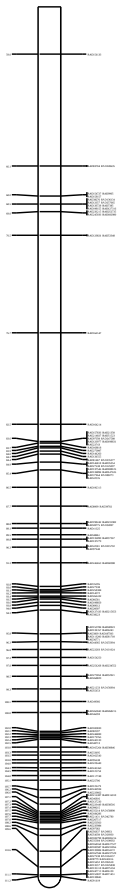

Catfish Genetic Map

27 [1]

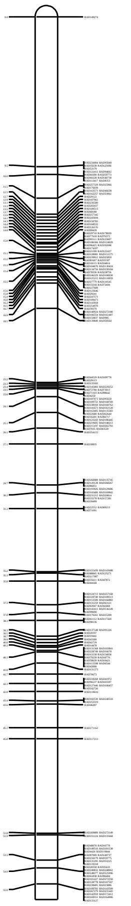

Catfish Genetic Map

27 [2]

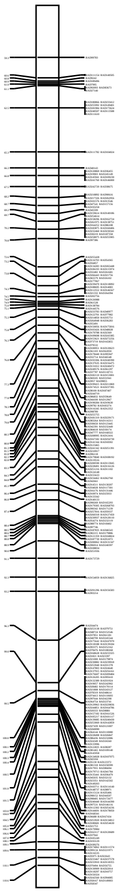

Catfish Genetic Map

27 [3]

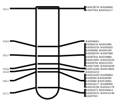

Catfish Genetic Map

28 [1]

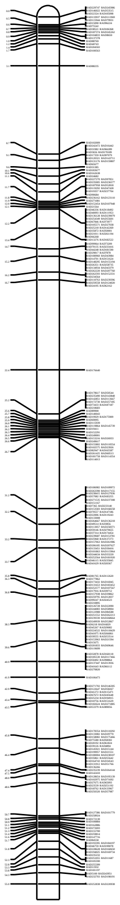

Catfish Genetic Map

28 [2]

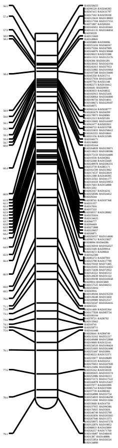

Catfish Genetic Map

29 [1]

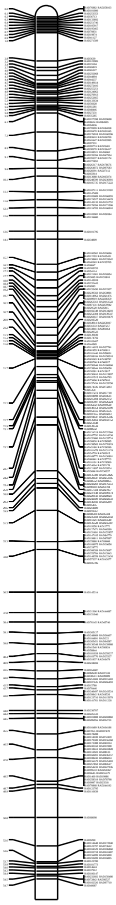

Catfish Genetic Map

29 [2]

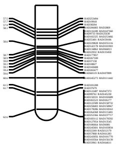

Supplement: Additional file 6 [file rsos172054supp7.pdf]
